# Negative automatic thoughts mediate the effects of emotion regulation on distress in women with breast and gynecological cancer

**DOI:** 10.1038/s41598-026-55214-4

**Published:** 2026-05-29

**Authors:** Katarina Bánfi, Róbert Urbán, Gyöngyi Kökönyei, Bernadette Kun, Gabriella Vizin

**Affiliations:** 1 Doctoral School of Psychology, ELTE Eötvös Loránd University, Budapest, Hungary; 2 Institute of Psychology, ELTE Eötvös Loránd University, Budapest, Hungary

**Keywords:** Emotion regulation, Distress, Automatic thoughts, Mediation, Cancer, Diseases, Health care, Psychology, Psychology

## Abstract

Cancer’s emotionally taxing nature offers a compelling real-world model for examining psychological distress. Despite its relevance, empirical research remains limited on the cognitive mechanisms, particularly self-referent negative automatic thoughts (NATs) and emotion regulation (ER), sustaining distress in chronic illness. In this cross-sectional study women (*N* = 230) diagnosed with breast or gynecological cancer completed self-report measures of distress, depressive and anxiety symptoms, cognitive emotion regulation and negative automatic thoughts, along with sociodemographic and clinical data. Structural equation modeling was used for analysis. Mediation analyses revealed that maladaptive emotion regulation was positively associated with psychological distress (*β* = 0.405), depression (*β* = 0.631), and anxiety (*β* = 0.660), with NATs partially mediating these effects. Adaptive ER showed weaker total effects, but significant indirect effects via reduced NATs. A latent distress or general psychological distress (PD) model demonstrated excellent fit and confirmed partial mediation. NATs uniquely predicted depressive symptoms beyond general distress, underscoring their central role. The findings highlight the central role of maladaptive ER and NATs in psychological distress among cancer patients. The results support cognitive models suggesting that targeting maladaptive cognitive patterns may yield improvements in emotional well-being. The PD model offers a parsimonious, transdiagnostic framework for conceptualizing shared vulnerability across symptom domains.

**Supplementary Information:**

The online version contains supplementary material available at 10.1038/s41598-026-55214-4.

## Introduction

Living with cancer involves a distinct psychological burden, characterized by ongoing uncertainty and existential threat. In 2022 alone, nearly 20 million individuals worldwide were diagnosed with cancer^1^, making it one of the most prevalent and emotionally taxing health conditions^2^. In the present study, we focus specifically on women with breast and gynecological cancers, which together account for the majority of cancer diagnoses in women and are associated with substantial long-term psychosocial burden^1,3,4^. Cancer-related psychological distress encompasses anxiety, sadness, helplessness, and fear, and may negatively impact quality of life, treatment adherence, and health outcomes^3,5–7^. Breast and gynecological cancers frequently face body-image–related and sexuality-related challenges and often involve prolonged treatment trajectories, including extended adjuvant therapies and long-term follow-up, which contribute to sustained emotional vulnerability in affected women^3^. This high-stakes, real-world context provides a contextually grounded framework for examining how cognitive mechanisms, such as emotion regulation and negative automatic thoughts, contribute to psychological distress.

Although cognitive therapy is well established as an evidence-based treatment for emotional disorders^8,9^, certain foundational constructs, notably negative automatic thoughts (NATs), remain underexplored as mediating factors in real-life adversity. NATs are involuntary, repetitive, self-referential cognitions that distort perception and amplify vulnerability^10,11^. While central to cognitive models of depression, these thought patterns have received limited empirical attention in broader stress contexts, such as chronic illness^12,13^. Recent conceptual work has further framed NATs as mental habits, automated, cue-triggered processes that can persist outside awareness and formal diagnosis^14^. Although negative automatic thoughts are central to cognitive models of emotional disorders and are amenable to change through cognitive-behavioral interventions, their role in the regulation of psychological distress under chronic stress conditions, such as cancer-related adversity, remains insufficiently investigated. This empirical gap is particularly striking given the theoretical emphasis on NATs as key mechanisms in the maintenance of distress and the clinical imperative to target distress symptoms in high-stakes, real-world contexts. The present study addresses this gap by examining the function of NATs within the cognitive-affective processes that shape emotional responses to chronic illness.

General psychological distress (PD), often understood as a transdiagnostic manifestation of psychological strain, serves as a clinically relevant proxy for cancer-related adversity^15^. Characterized by persistent negative affects (e.g., anxiety, sadness, irritability), cognitive disruption (e.g., intrusive thoughts, attentional biases), behavioral withdrawal, and autonomic dysregulation, general PD typically emerges when coping resources are exceeded^16^. Consistent with higher-order models of distress showing that anxiety, depressive affect, and related symptoms load onto a common general distress factor^17^, substantial empirical overlap among these domains has also been documented in primary care^18^ and in oncology populations^19^. Accordingly, general PD is frequently conceptualized as a broad, higher order construct rather than a set of discrete symptom domains^15,17^. It is particularly relevant in cancer, where individuals often report significant general psychological distress despite not meeting diagnostic thresholds for psychiatric disorders^20,21^. Emotion regulation strategies (ERS) significantly shape individuals’ responses to stress and trauma and are increasingly recognized as transdiagnostic processes that influence psychological functioning across a wide range of mental health conditions^22^. Emotion regulation encompasses a multifaceted system involving physiological responses, conscious and unconscious cognitive mechanisms, and a wide range of social and behavioral coping strategies^23^, however, the present study focuses specifically on conscious cognitive emotion regulation strategies. Across diverse populations, maladaptive cognitive strategies, such as catastrophizing, self-blame, and rumination, have been consistently linked to heightened psychological vulnerability, whereas adaptive approaches like positive reappraisal and perspective-taking serve as protective mechanisms that buffer psychological distress^22–24^. Empirical research has demonstrated robust associations between specific ERS and emotional outcomes, with maladaptive strategies showing transdiagnostic relevance across various forms of psychopathology^22,25^. Longitudinal and clinical data further suggest that successful application of ER skills may be particularly important for the treatment and maintenance of depressive symptoms, as evidenced in studies on individuals with major depressive disorder^26^ and adolescents from the general population^27^. Regulatory patterns such as suppression, acceptance, and reappraisal have also been shown to modulate both physiological and subjective anxiety responses^28^.

Drawing from these general findings, a growing body of evidence highlights the relevance of ERS in chronic stress contexts, particularly in medical chronic disease^29,30^ and cancer in specific^31^. Studies involving cancer patients and survivors suggest that emotion regulation is especially salient in the face of cancer-related adversity, where distress is shaped not only by external stressors but by how individuals manage their emotional responses^29,31^. Maladaptive strategies like rumination have been found to predict elevated distress following cancer diagnosis^32^, while higher reappraisal has been linked to higher engagement in health-promoting behaviors, such as physical activity^33^. Among cancer patient populations, maladaptive cognitive emotion regulation strategies (such as self-blame, rumination, and catastrophizing) have been associated with reduced quality of life, whereas adaptive strategies like acceptance and positive reappraisal demonstrate beneficial effects^34,35^. Together, these findings underscore emotion regulation as a pivotal mechanism underlying psychological distress, particularly under conditions of prolonged stress such as chronic illness.

Building on recent evidence that emotion regulation strategies function as transdiagnostic processes across psychological disorders^22^ and may influence distress outcomes in cancer populations^32^, emerging research suggests that these effects may be mediated by negative, biased cognitive contents, particularly negative automatic thoughts^36^. Maladaptive emotion regulation strategies, such as rumination, catastrophizing, or self-blame, can trigger and sustain negative thinking patterns and reinforce psychological distress^23^. For instance, catastrophizing may lead individuals to interpret minor symptoms as signs of illness recurrence, while self-blame can generate persistent thoughts of personal failure or guilt. Rumination perpetuates psychological distress by repeatedly sustaining attention on negative appraisals^37^ showing impaired ability to disengage from maladaptive thought patterns^38^ and may hinder the use of adaptive emotion regulation strategies that on the other hand can promote psychological well-being^39^. While rumination and negative automatic thoughts are conceptually distinct, rumination being a repetitive cognitive process and NATs its often negatively biased content, they are closely intertwined, and their dynamic interaction warrants further empirical investigation^40,41^. This underscores the need for further empirical exploration of the relationship between ER and biased cognitive content (NATs) in influencing emotional outcomes, positioning NATs as a possible key mediator between emotion regulation and psychological outcomes. However, integrative models that formally test these pathways in oncology populations are still scarce.

The present study aimed to examine how cognitive emotion regulation strategies and negative automatic thoughts are associated with psychological distress in women diagnosed with breast or gynecological cancer. While prior research has demonstrated the individual contributions of ER strategies and NATs to psychological distress, few studies have integrated these constructs into a unified model in the context of oncology. We addressed this gap by testing a model in which NATs partially mediate the relationship between ER strategies and distress. In formulating our model, we drew on cognitive theories of depression, which emphasize that a key vulnerability factor is a cognitive style marked by frequent, automatic, negative self-referential thoughts^42,43^. Emotion regulation difficulties, particularly ruminative processing, also represent a well-established risk factor for the onset and maintenance of depression^37,41^. Importantly, prior work suggests that not all forms of rumination are equally implicated; rather, maladaptive brooding, characterized by repetitive self-referent negative thinking, plays a central role in sustaining depressive symptoms^44^. Neurocognitive research further indicates that failures in cognitive reappraisal and heightened rumination are linked to emotion dysregulation through mechanisms such as impaired default mode network modulation of self-referential processing^45^. Phenomenologically, these processes hypothetically manifest as an increased presence of negative automatic thoughts, positioning NATs as the proximal cognitive expressions of dysregulated affect. At the same time, we acknowledge that alternative conceptualizations are also theoretically plausible^22,43,46^. Negative automatic thoughts may not only arise from maladaptive emotion regulation processes^10,11,46^ but may also shape subsequent regulatory responses by narrowing attention, reinforcing threat- or loss-related appraisals, and making adaptive strategies such as reappraisal or perspective-taking more difficult to implement^47^. In this view, NATs may contribute to the persistence of maladaptive strategies such as rumination, catastrophizing, and self-blame^48^. Thus, although the present study primarily tested an ER → NATs → distress pathway grounded in cognitive-behavioral and emotion regulation theories, we also considered the theoretically plausible reversed pathway, NATs → ER → distress^49^, as a sensitivity analysis given evidence that differentiating cognition, emotion, and cognitive emotion-regulation processes is inherently difficult^48^.

In line with a multidimensional conceptualization of psychological distress, we assessed depressive symptoms, anxiety, and cancer-distress as core indicators. Analytically, these outcomes were examined both as separate observed variables and as indicators of a latent PD factor, allowing us to test both specific and generalized patterns of psychological strain. Given the substantial empirical overlap among anxiety, depressive affect, and cancer-related distress in oncology populations, modeling a latent factor provides a theoretically grounded way to capture their shared variance and to examine general psychological distress as a higher order construct rather than a single symptom domain^15,17,50^.

**Fig. 1:**
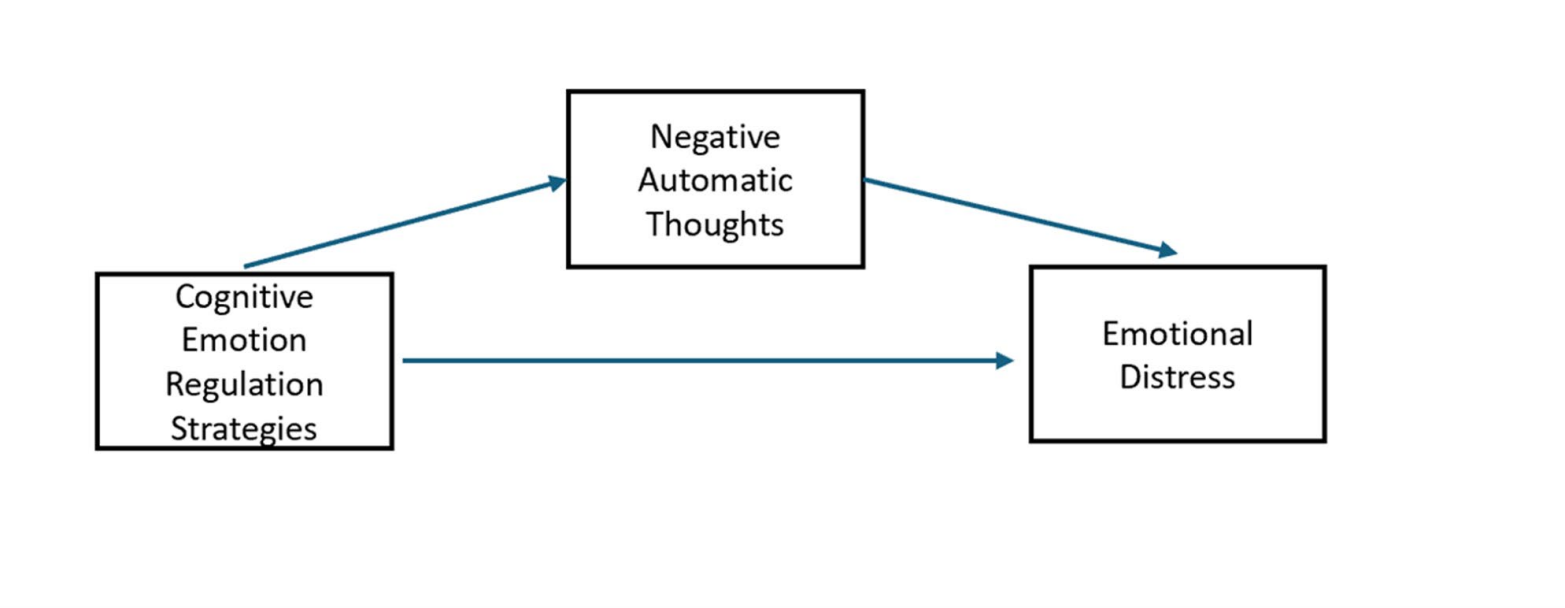
Hypothesized conceptual model of the study.

This study tested a model in which NATs mediate the link between cognitive emotion regulation and distress in women with breast or gynecological cancer, contributing to a more integrative understanding of the psychological mechanisms underlying cancer-related emotional burden in this population. Consistent with prior research^36^, we expect only partial mediation because emotion regulation strategies have been shown to influence distress both directly and indirectly through cognitive processes such as negative automatic thoughts. Thus, NATs were conceptualized as one pathway through which emotion regulation contributes to psychological distress, rather than a mechanism that fully accounts for this association. Figure 1 illustrates the hypothesized relationships between emotion regulation strategies, negative automatic thoughts, and distress. In addition to the primary theory-driven model, we estimated theoretically plausible reversed models (NATs → ER → distress)^49^ as sensitivity analyses to examine whether the proposed directional structure was uniquely supported by the data.

Based on previous literature, we formulated the following hypotheses:

1. Higher levels of maladaptive emotion regulation strategies (e.g., rumination, catastrophizing, self-blame) will be associated with higher levels of psychological distress, while higher levels of adaptive strategies (e.g., reappraisal, acceptance) will be associated with lower levels of distress.
2. A greater frequency of negative automatic thoughts will be associated with higher levels of psychological distress.
3. Negative automatic thoughts will be associated with greater depressive symptoms, even after controlling for psychological distress.
4. Negative automatic thoughts will partially mediate the relationship between maladaptive emotion regulation strategies and psychological distress.

## Results

### Sample inclusion and missing data handling

Among all cancer patients who completed the questionnaire battery, a total of 313 female respondents diagnosed with breast or gynecological cancer were selected for inclusion in the present study. Participants with missing data on the main explanatory variables were excluded prior to model estimation because these variables were treated as exogenous covariates in the SEM models. Because exogenous predictors were treated as fixed rather than random variables, missing data on these variables could not be handled within the FIML framework^51^. For the remaining variables in the model, missing data were handled using Full Information Maximum Likelihood (FIML). As a result, the final analytic sample consisted of 230 individuals. Participants included in the analysis did not differ significantly from those excluded in terms of available data on age, distress (DT), depressive symptoms (PHQ-9), or anxiety symptoms (GAD-7). However, those who discontinued the study early and were excluded from the analysis were more likely to be in a less advanced stage of cancer (80.7% vs. 66.1%; *χ*^2^ = 6.20, *p* = .013).

### Sample characteristics and descriptive statistics

The final analytic sample included 230 female cancer patients. Among them, 160 (69.6%) were diagnosed with breast cancer, 34 (14.8%) with ovarian cancer, 11 (4.8%) with uterine cancer, and 25 (10.9%) with cervical cancer. In total, 79 participants (33.9%) were classified as being in an advanced stage of cancer. Participants ranged in age from 27 to 82 years (*M* = 52.18, *SD* = 10.06).

Participants’ educational levels ranged from eight-year elementary schooling (2.6%) and vocational secondary education (13%) to doctoral-level education (4.8%). Half of the sample (50%) held a higher education degree, and the majority (75.7%) resided in urban areas compared to rural settings. In terms of marital status, most participants (65.2%) were married or cohabiting, and 76.5% reported having children.

Regarding medical data, cancer stages among participants ranged from 0 to IV. A majority (61.8%) were in the early stages of the disease (stages 0–II or in remission), while 33.9% were diagnosed at advanced stages (III–IV). Surgical intervention was performed in 86.5% of cases: 53.5% underwent a single procedure, while 33% had multiple surgeries. Approximately half of the participants (49.6%) received radiotherapy, and nearly the same proportion (44.3%) were undergoing hormonal therapy at the time of data collection. For detailed statistics, see Table 1.

**Table 1:** Descriptive characteristics of study sample.

Characteristic	No. of participants	%
*N*	230	
Marital status		
Married or cohabitating	169	73.5
Divorced or widowed	27	11.7
Single	24	10.4
Other	10	4.4
Education		
< High school	36	15.6
High school	79	34.3
College/University graduate	115	50.0
No. of children		
0	54	23.5
1	63	27.4
2	78	33.9
≥ 3	35	15.2
Type of residence place		
Urban area	174	75.7
Rural area	56	24.3
Cancer diagnoses		
Breast cancer	160	69.6%
Ovarian cancer	34	14.8%
Cervical cancer	25	10.9%
Uterine cancer	11	4.8%
Stage		
0	9	3.9
I	48	20.9
II	51	22.2
III	52	22.6
IV	26	11.3
Remission	9	3.9
“I don’t know”	25	10.9
Medical treatment		
Surgery	199	86.5
No surgery	29	12.6
One surgery	123	53.5
> 1 surgery	76	33.0
Radiotherapy	114	49.6
Hormonal therapy	102	44.4
Immunotherapy	19	8.3
Other	27	11.7

Table 2 summarizes the descriptive statistics, internal consistency estimates, and intercorrelations among the main study variables. The psychological measures demonstrated acceptable to excellent internal consistency. As expected, distress showed moderate-to-strong positive associations with depressive symptoms, anxiety, automatic negative thoughts, and maladaptive emotion regulation, while it was negatively related to adaptive regulation strategies. Negative automatic thoughts were strongly linked to both emotional symptoms and maladaptive regulation, highlighting their central role in emotional vulnerability. Adaptive and maladaptive regulation strategies were modestly but significantly inversely correlated. For correlation coefficients among cognitive emotion regulation subscales (CERQ-18) see Supplementary Table S1.

**Table 2:** Descriptive statistics, internal consistencies, and pearson’s correlations between study variables.

	1.	2.	3.	4.	5.	6.	7.	8.	9.
1. Distress (DT)									
2. Depression symptoms (PHQ-9)	0.589***								
3. Anxiety symptoms (GAD-7)	0.623***	0.783***							
4. Automatic thoughts (ATQ-8)	0.453***	0.710***	0.662***						
5. Adaptive emotion regulation (CERQ-18)	–0.170**	–0.214**	–0.169*	–0.240***					
6. Maladaptive emotion regulation (CERQ-18)	0.428 ***	0.651 ***	0.680 ***	0.727 ***	–0.185**				
7. Age	–0.026	–0.046	–0.041	–0.040	–0.096	–0.078			
8. After treatment^#1^	–0.073	0.002	0.055	0.030	0.012	0.004	0.101		
9. Before treatment^#2^	0.099	0.116	0.195**	0.111	–0.021	0.169*	–0.06	0.142*	
10. Cancer stage^#3^	0.044	–0.003	–0.079	0.070	–0.066	0.012	0.071	–0.336***	–0.05
Scale range	0–10	0 − 7	0–21	8–40	10–50	8–40			
Mean	5.93	18.14	14.49	17.03	31.11	15.54			
*SD*	3.10	5.99	5.24	6.75	6.47	5.47			
Cronbach’s *α* McDonald’s ω	n/a n/a	0.87 0.87	0.91 0.91	0.91 0.91	0.77 0.77	0.83 0.83			

*N* = 230; *p < .05; **p < .01; ***p < .001.

SD, standard deviation; DT, Distress Thermometer; PHQ-9, Patient Health Questionnaire–9; GAD-7, Generalized Anxiety Disorder–7; ATQ-8, Automatic Thoughts Questionnaire, short form; CERQ-18, Cognitive Emotion Regulation Questionnaire, short form.

# Variables were dummy coded: Treatment status – Before treatment #2 (1 = yes, 0 = otherwise), After treatment #1 (1 = yes, 0 = otherwise); Cancer stage – (1 = stage III or IV, 0 = stage 0, I, II, remission, or “do not know”).

### Mediation analysis: two alternative models

**Fig. 2:**
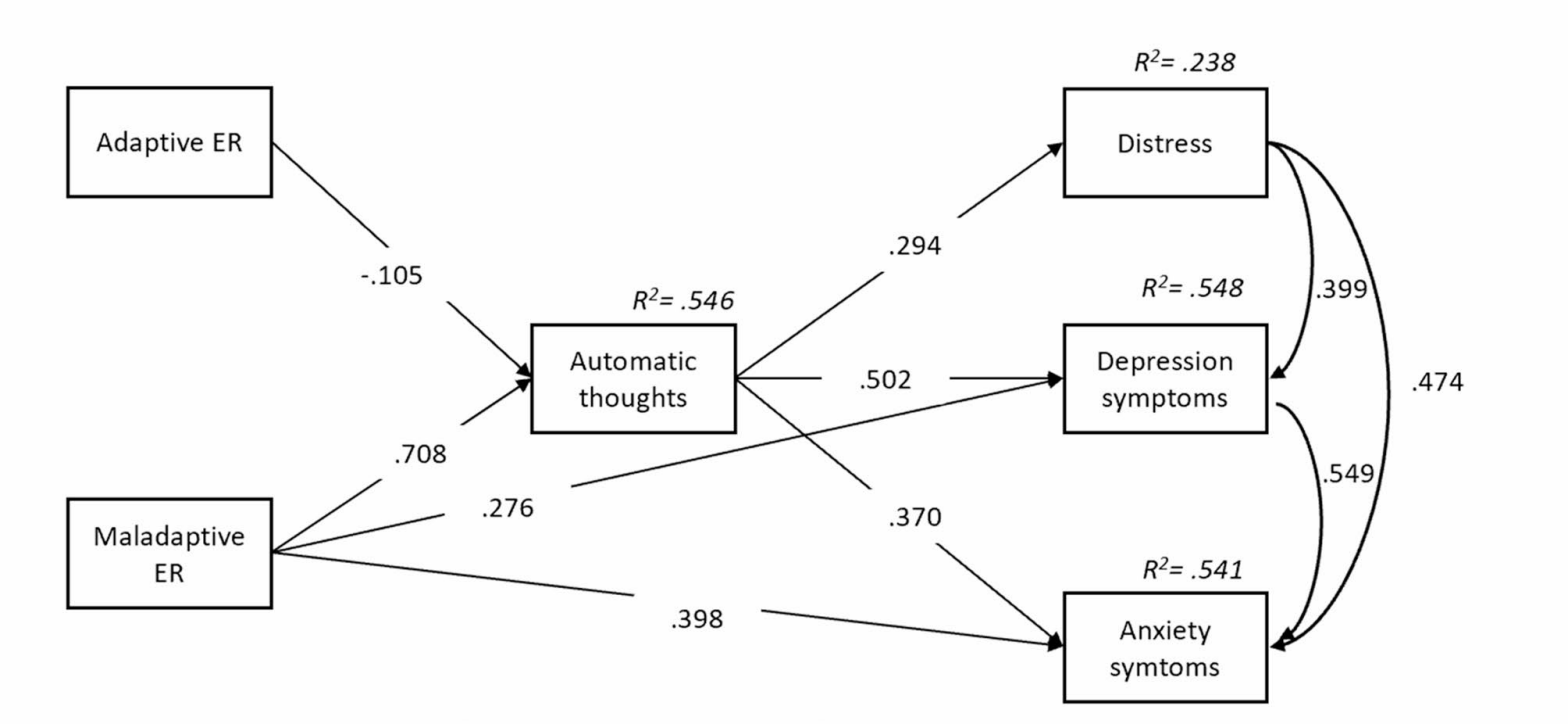
The path model (Model 1) presenting standardized, significant (*p* < .05) path coefficients among variables.

Figure 2 presents the tested path model (see Supplementary Table S2b for detailed report of standardized total, indirect, and direct effects) examining the relationships among emotion regulation strategies, negative automatic thoughts, and psychological distress outcomes. In this model, maladaptive emotion regulation was positively associated with automatic thoughts, whereas adaptive emotion regulation showed a weak negative association with automatic thoughts. Automatic thoughts, in turn, were significantly related to distress, depressive symptoms, and anxiety symptoms.

The mediation analysis further clarified these relationships. Specifically, maladaptive emotion regulation showed significant total effects on distress (*β* = 0.405, *p* < .001), depressive symptoms (*β* = 0.631, *p* < .001), and anxiety symptoms (*β* = 0.660, *p* < .001). These effects were partly mediated by negative automatic thoughts, with significant indirect effects on distress (*β* = 0.209, 95% CI [0.082, 0.340], *p* = .002), depressive symptoms (*β* = 0.356, 95% CI [0.249, 0.492], *p* < .001), and anxiety symptoms (*β* = 0.262, 95% CI [0.160, 0.374], *p* < .001). Notably, the direct effects of maladaptive emotion regulation on depressive and anxiety symptoms remained significant (*β* = 0.276, *p* < .001 and *β* = 0.398, *p* < .001, respectively), suggesting partial mediation.

In contrast, adaptive emotion regulation exhibited weaker relationships. The total effects on distress (*β* = − 0.092, *p* = .184), depressive symptoms (*β* = − 0.099, *p* = .054), and anxiety symptoms (*β* = − 0.049, *p* = .346) were nonsignificant or marginal. However, significant indirect effects through negative automatic thoughts were observed for depressive (*β* = − 0.053, 95% CI [–0.015, − 0.104] *p* = .022) and anxiety symptoms (*β* = − 0.039, 95%CI [–0.009, − 0.085] *p* = .045), indicating that adaptive strategies may reduce these symptoms primarily via lowering automatic negative thoughts rather than through direct pathways.

Age, treatment status (dummy-coded), and cancer stage were statistically controlled for in the model but are not displayed in the figure for clarity. As the model was fully saturated, traditional model fit indices were not evaluated, with the exception of the sample-size adjusted Bayesian Information Criterion (ssaBIC), which is used for model comparison purposes for non-nested models. The ssaBIC value was 4860.8.

**Fig. 3:**
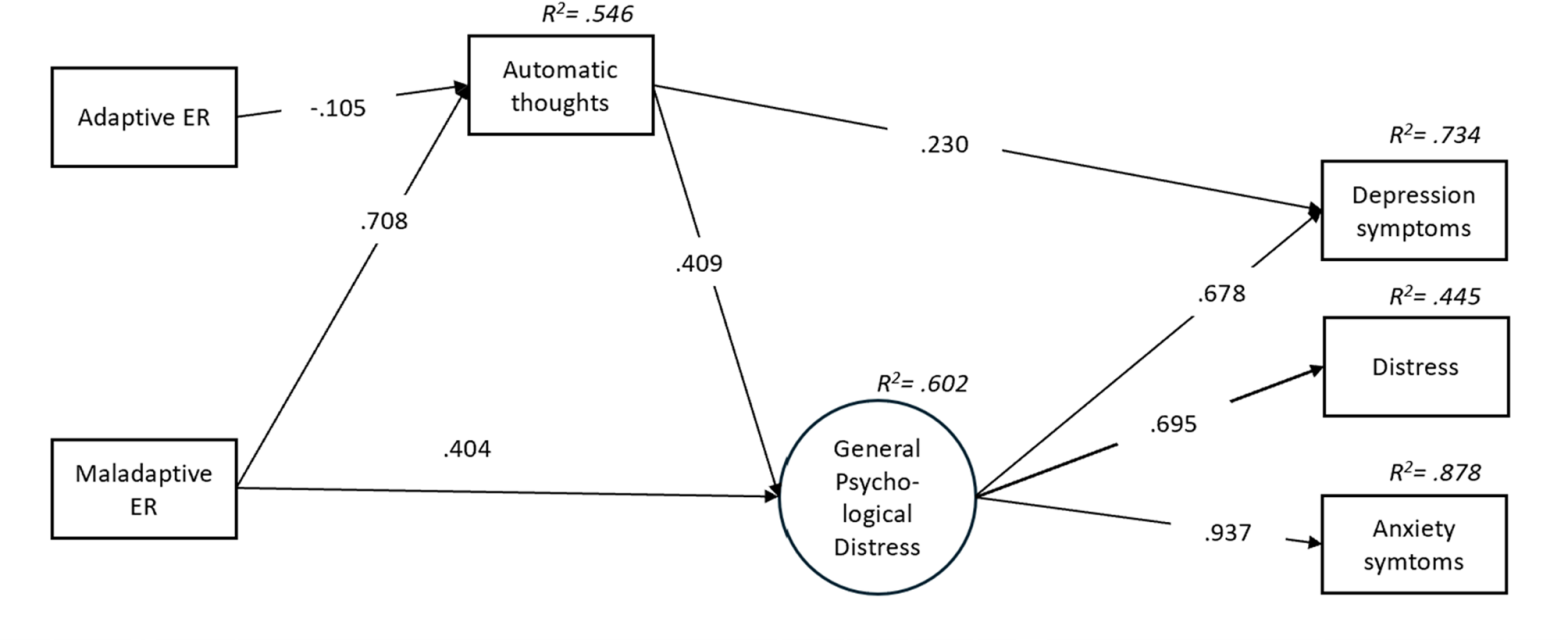
The alternative path model (Model 2) presenting only standardized, significant (*p* < .05) path coefficients among variables.

Figure 3 presents an alternative path model (see Supplementary Table S3b for detailed report of standardized total, indirect, and direct effects) in which distress, depressive symptoms, and anxiety symptoms were specified as indicators of a single latent factor representing general psychological distress. This model showed excellent fit to the data (*χ²* (12) = 14.08, *p* = .296; RMSEA = 0.027 [90% CI: 0.000, 0.075], CFI = 0.997, TLI = 0.993, SRMR = 0.026, ssaBIC = 4847.6), supporting the validity of the latent distress factor approach.

In this model, maladaptive emotion regulation demonstrated significant total (*β* = 0.693, *p* < .001) and direct effects (*β* = 0.404, *p* < .001) on the latent distress factor. A significant indirect effect via negative automatic thoughts was also observed (*β* = 0.290, 95% CI [0.173, 0.403], *p* < .001), indicating partial mediation.

Adaptive emotion regulation exhibited a nonsignificant total effect on the latent distress factor (*β* = − 0.072, *p* = .212). However, a significant indirect effect through automatic thoughts was identified (*β* = − 0.043, 95% CI [–0.091, − 0.010], *p* = .040), suggesting that adaptive strategies reduce distress primarily by decreasing negative automatic thoughts rather than via a direct pathway (direct effect: *β* = − 0.029, *p* = .593).

Additionally, beyond their role in explaining general psychological distress, negative automatic thoughts had a unique direct association with depressive symptoms even after accounting for the latent distress factor. This pattern highlights that automatic thoughts not only contribute to a general vulnerability to psychological distress but also play a particularly central role in depressive symptoms specifically. In contrast, no such unique direct association was observed with anxiety symptoms beyond their shared variance captured by the latent factor.

Furthermore, the latent distress model was retained as a theoretically motivated and more parsimonious representation of the outcome domain, rather than based on a direct comparison of conventional fit indices with the saturated observed-outcome model. Specifically, this model captures the shared variance among cancer-related distress, depressive symptoms, and anxiety symptoms as a general psychological distress factor, while also allowing symptom-specific residual associations to be examined. The lower sample-size- adjusted Bayesian Information Criterion (ssaBIC) value of the latent model was therefore interpreted only as supplementary descriptive information, rather than as a primary basis for model selection. Additionally, the theoretically plausible reversed models (NATs → ER → GPD) yielded comparable fit indices, as detailed in Supplementary Tables S4–S5 and S6 indicating that the preferred directional structure is supported primarily on theoretical rather than empirical grounds.

## Discussion

Measuring, understanding, and addressing depression, anxiety, and distress are critical priorities in the psychosocial care of cancer patients^20^ and individuals with chronic disease more broadly^52^ as they play a pivotal role in facilitating the complex process of psychological adjustment^29,53^. Building on this clinical imperative, the present study tested a mediation model grounded in the cognitive theory of depression^12,42^, to examine how self-referent negative automatic thoughts and emotion regulation processes are linked to psychological distress in the context of chronic illness-related stress.

To enhance methodological clarity and explore the structure of psychological distress^55^, we compared two competing models: one treating depression, anxiety, and cancer-related distress as distinct but correlated constructs, and another modeling their shared variance as a single latent factor representing general psychological distress^15,17^. This comparison allowed us to examine whether a unified latent general psychological distress construct better captures the underlying cognitive-emotional dynamics in the context of chronic stress. Psychometric studies have also presented evidence for a strong general PD factor and relatively weak specific depression and anxiety factors when examining the factor structures of specific scales (e.g., Hospital Anxiety and Depression Scale^56,57,59^. However, our study included three distinct indicators of depression, anxiety, and distress. The advantage of including a latent general distress factor is that it not only integrates common emotional vulnerability but also allows for the estimation of specific mechanisms for each outcome while controlling for this shared component. Importantly, because the observed-outcome model was fully saturated, fit indices could not be used as a meaningful basis for model selection^53^. The latent distress model was therefore retained on theoretical and parsimony grounds, as it captures the shared variance among depression, anxiety, and cancer-related distress.

The latent distress specification is consistent with extensive latent variable research demonstrating that common mental health measures, particularly depression and anxiety, tend to load onto a general psychological distress dimension rather than forming fully independent constructs^17,55-57^. This approach is particularly relevant for oncology patients, who often experience overlapping emotional difficulties rather than discrete psychiatric syndromes; thus, focusing on a shared latent general PD factor may facilitate transdiagnostic interventions tailored to their complex psychological needs^60^. Addressing a gap in literature^49^, this study examined how cognitive emotion regulation strategies and negative automatic thoughts jointly contribute to psychological distress, extending prior research that has predominantly treated these constructs separately. As noted in the introduction, few studies^36,49^ have integrated these mechanisms into a unified model, particularly within the real-world contexts of chronic stress. By leveraging a high-stress, authentic real-life setting, this research offers novel insight into how regulatory processes and cognitive patterns interact to contribute to psychological strain. Modeling NATs as a partial mediator and assessing both specific symptoms and a latent distress factor allowed for a multidimensional understanding of vulnerability under prolonged stress exposure.

Our results also emphasize the central role of maladaptive emotion regulation in depression, anxiety, and distress, through its strong direct and indirect effects on all PD outcomes. This finding is consistent with other studies reporting that maladaptive emotion regulation strategies are positively correlated with depression in cancer patients^61^. Clinically, this highlights the importance of targeting maladaptive strategies, such as rumination^58^, as key intervention points. Rather than solely promoting adaptive techniques like positive reappraisal or problem-solving, reducing maladaptive patterns may be particularly critical for achieving meaningful improvements in psychological well-being^58^. This is especially relevant in oncology, where patients often rely on maladaptive emotion regulation strategies, which can exacerbate distress over time. Tailored interventions focusing on reducing maladaptive emotion regulation may thus offer a powerful means to mitigate emotional suffering and improve overall adjustment during cancer treatment^58^.

Our findings highlight that adaptive emotion regulation strategies might play a more subtle yet meaningful role in psychological outcomes among oncology patients^64^. While their direct effects on distress, depression, and anxiety symptoms were weak or nonsignificant, significant indirect associations still suggest a beneficial association. This underscores that adaptive strategies can support psychological well-being^39^, even if their associations are less immediate or pronounced than those of maladaptive strategies, a pattern also reflected in meaning-making research with cancer survivors^65^. Clinically, promoting adaptive emotion regulation remains important for improving overall adjustment during cancer treatment^61,64,65,89^.

Our findings also reveal important role of negative automatic thoughts^36,62^ in the psychological adjustment of oncology patients. Beyond mediating the effects of emotion regulation strategies on latent general PD, NATs also demonstrated a unique and specific direct association with depressive symptoms^13,42^, even after controlling for their shared variance with general distress. This suggests that NATs contribute not only to a vulnerability to emotional suffering but play a particularly important role in shaping depressive experiences^42,43,44^. Importantly, these findings support a hierarchical conceptualization in which emotion regulation strategies reflect broader vulnerability processes^24,27,63^, while negative automatic thoughts represent more proximal cognitive mechanisms^13,42,44,45^ through which these vulnerabilities manifest in distress. In this framework, NATs may function not merely as correlates but as active mediating processes^36,45^, consistent with Beck’s cognitive model of depression^42^, which posits that persistent, pervasive negative automatic thoughts might be a defining feature of depression and may act as a core maintaining factor^13,42,62^.

Clinically, this underscores the well‑established role of targeting NATs in depression among cancer patients, a relationship extensively supported by cognitive‑behavioral research showing that modifying NATs leads to meaningful reductions in depressive symptoms^8,58,64,65^. While general approaches to distress management may address shared emotional vulnerabilities, focused cognitive interventions that explicitly challenge and modify these automatic thoughts may be essential to achieving significant improvements in depressive symptoms. These results highlight the potential added value of integrating cognitive restructuring techniques alongside emotion regulation strategies in psycho-oncological care^66–69,71^. In the oncology context, positive reframing may be most effective when embedded within broader meaning-making processes that help patients integrate and reconcile meaning discrepancies arising from illness-related adversity^64,65,69^. This approach can facilitate a cognitive shift away from pervasive ruminative, catastrophic, and self- or other-blaming thought patterns toward more adaptive interpretations that support adaptive behavioral coping and psychological adjustment^31,54,65,66^. Early assessment of NAT patterns may further enhance clinical outcomes by enabling timely intervention and potentially preventing the escalation of subclinical distress into major depressive episodes.

## Limitations and future directions

This study has several limitations that should be acknowledged. First, its cross-sectional design precludes any conclusions about temporal ordering or causal relationships. Accordingly, the mediation pathways tested in this study should be interpreted as theory-driven^11,13^ statistical associations among cognitive-emotional processes rather than as evidence of causal mechanisms. Although classical cognitive-behavioral frameworks^10,42^ guided our theoretical model, it is possible that psychological distress, anxiety, and depressive symptoms may in turn contribute to increases in negative automatic thoughts and reinforce maladaptive emotion regulation strategies, rather than the other way around. The comparable fit of the theoretically plausible reversed models further supports this cautious interpretation. Evidence from other clinical populations supports the temporal precedence of automatic thoughts over depression; for example, in people living with HIV/AIDS, reductions in negative automatic thoughts were shown to precede reductions in depressive symptoms, suggesting that targeting cognitive patterns can drive mood improvement rather than merely reflect it^62^. Second, data were collected using self-report measures administered online. Such measures are inherently subject to recall bias and potential distortion due to social desirability. While recall bias cannot be eliminated, the anonymous online environment may have helped reduce social desirability effects. Third, the study employed a convenience sampling approach, introducing self-selection bias. Individuals who chose to participate were likely more open to reflecting on and disclosing their psychological difficulties and were also comfortable using online platforms. This may have led to an overrepresentation of participants with higher education levels and greater digital literacy. Additionally, disease severity may have influenced willingness to participate; notably, patients with more advanced cancer stages appeared more likely to complete the relatively lengthy questionnaire. Fourth, emotion regulation was assessed solely using the CERQ, which captures selected cognitive strategies but excludes other relevant forms such as behavioral avoidance and emotional suppression. Additionally, it remains unclear whether participants interpreted the CERQ instructions in a general context or specifically in relation to cancer-related stress, potentially affecting response validity. A further limitation is that, although the Hungarian versions of the ATQ-8, PHQ9- and GAD-7 are used in research, formal validation studies of these translations are not yet available, and their use as latent SEM indicators should therefore be interpreted with appropriate caution. Fifth, although maladaptive emotion regulation and negative automatic thoughts were strongly correlated (*r* = .727), this association did not indicate severe multicollinearity based on conventional diagnostics (e.g., VIF = 2.12). Nevertheless, the substantial shared variance between these theoretically related constructs may have contributed to the magnitude of the observed indirect effects and therefore warrants cautious interpretation. Future research should further examine whether maladaptive emotion regulation, particularly rumination, catastrophizing, and self-blame, can be empirically distinguished from negative automatic thoughts, which may be understood as negative self-referential cognitive content.

An additional limitation is that, beyond the variables included in our model, numerous other factors may influence psychological outcomes. Some potentially relevant clinical variables could not be included or adequately controlled due to the small sample size, such as type of cancer or in particular time since first diagnosis, a factor known to influence psychological adjustment, which represents a notable limitation when interpreting the findings^72^. Future research would benefit from incorporating these factors to provide a more comprehensive understanding of distress in chronic stress contexts. Finally, the generalizability of our findings is limited to Hungarian female cancer patients diagnosed with breast or gynecological cancers. While this focus was chosen intentionally to reduce potential unmeasured confounding variables related to gender differences, caution should still be exercised when extending these results to other populations, including men, patients with other cancer types, or individuals from different cultural or healthcare contexts.

Besides these limitations, from a theoretical perspective, our results support the added value of a latent distress factor in simplifying the conceptualization of emotional difficulties and fostering a transdiagnostic framework. This approach emphasizes shared underlying mechanisms, such as negative self-referent cognitions and deficits in emotion regulation, which can serve as central targets for psychological interventions across diagnostic categories. At the same time, it is important to acknowledge that the relatively high R² values observed in our models may partly reflect shared method variance or conceptual overlap among these constructs, given the cross-sectional and self-report nature of the data. Overall, our findings highlight the potential value of integrating targeted interventions that focus on maladaptive emotion regulation and negative automatic thoughts to improve mental health outcomes and enhance quality of life among cancer patients.

Future research should utilize multi-method assessments and longitudinal designs to rigorously evaluate the distinct and shared contributions and clarify causal relationships and temporal dynamics among these cognitive-emotional processes^73^. Further studies should also include male patients and other cancer types to increase generalizability. Additionally, tracking the dynamic changes in cognitive^12,42,74^ and metacognitive^78^ factors during intervention studies could help clarify the directionality of these relationships and inform mechanisms of therapeutic effectiveness. The findings of our study underscore the clinical importance of developing and implementing targeted interventions that specifically enhance “more adaptive” emotion regulation and cognitive restructuring capacities. These transdiagnostic strategies could serve as foundational components in psycho-oncological care, offering scalable and effective means to reduce distress across diverse cancer populations.

## Conclusions

This study underscores the central role of maladaptive emotion regulation and negative automatic thoughts in shaping psychological distress among oncology patients. By modeling both specific symptoms and a latent distress factor, we demonstrate that transdiagnostic cognitive-emotional mechanisms, particularly maladaptive emotion regulation strategies and self-referent negative thinking, are critical targets for intervention. While adaptive regulation strategies showed modest effects, their indirect associations suggest value in promoting cognitive flexibility. Clinically, these findings support the integration of cognitive restructuring and emotion regulation training to reduce distress and depressive symptoms. Future research should further refine transdiagnostic models and evaluate tailored interventions in diverse cancer and possibly other chronic disease populations.

## Method

### Participants and procedure

Data for the present study were collected in Hungary between December 2024 and May 2025, as part of a larger, ongoing cross-sectional questionnaire-based research project focusing on people with cancer diagnosis. The study protocol received ethical approval from the Hungarian National Scientific and Research Council Ethical Review Board (Ref. No. BM/15042-1/2024) and was conducted in accordance with the principles of the Declaration of Helsinki. Participants were recruited through two primary channels. First, clinical referrals were made by healthcare personnel at the Oncology Department of the Semmelweis University Clinical Centre in Budapest, who distributed printed flyers and displayed posters in the clinic’s waiting area. Second, eligible individuals were invited via posts in social media groups affiliated with Hungarian cancer survivor support associations targeting women (e.g. Health Bridge Alliance Against Breast Cancer and Mallow Flower Foundation). Inclusion criteria were as follows: being female, aged 18 or older, having a diagnosis of breast or gynecological cancer, providing informed consent, and being proficient in the Hungarian language. The questionnaire battery was administered online and made accessible to participants via a hyperlink and QR code. Both data collection and the informed consent procedure were conducted using the Qualtrics survey platform and required approximately 20–25 min to complete. Prior to participation, individuals received detailed information about the research project, including the study’s purpose and data management procedures. Participants were not offered any material incentives for taking part in the study.

Completion of the demographic, diagnostic, treatment-related questions and psychological scales was permitted only after participants had provided informed consent. Participation was entirely voluntary, no material incentives were offered, and respondents could discontinue their participation at any time without consequences. In addition, given the clinical nature of the sample and the sensitivity of the topic, participants were informed that they could contact the research team for support or consultation if they experienced emotional discomfort during the study.

The current analysis is based on a convenience sample of 230 self-identified female participants (*M*_age = 52.18, *SD* = 10.06) diagnosed with either gynecological or breast cancer. From the initial dataset of 609 entries, cases were excluded due to duplicate submissions identified via IP address and response patterns (*n* = 76, 12.5%), unrealistically short completion times (< 100 s; *n* = 120, 19.7%), very low response completeness (< 10% of items completed; *n* = 24, 3.9%), and failure to meet the inclusion criteria (*n* = 76, 12.5%). After data cleaning, 313 eligible women diagnosed with breast or gynecological cancer remained. Of these, 230 had complete data on the explanatory variables and were included in the final analyses (see Statistical Analysis for details on missing data handling). For the SEM analyses, participants with missing data on exogenous predictors were excluded because these variables were treated as fixed covariates. As a result, the final analytic sample consisted of 230 individuals. Missing data on endogenous variables were handled using Full Information Maximum Likelihood (FIML).

### Measures

#### Distress

The *National Comprehensive Cancer Network Distress Thermometer (NCCN DT)*^5,6^ is a widely used screening tool^5^ that assesses overall psychological distress experienced during the past week, including the current day. It consists of a single-item 11-point visual analog scale ranging from 0 (“no distress”) to 10 (“extreme distress”). Distress is defined as a negative mental, physical, social, or spiritual state that hinders coping with cancer^75,76^. The DT is designed to be a quick and effective method to identify patients who may benefit from further psychosocial support, and it has been validated in numerous oncological populations^75,76^.

#### Depressive symptoms

*The Patient Health Questionnaire–9 (PHQ-9)* developed by Kroenke et al.^77^, is a 9-item self-report instrument applied to assess the severity of depressive symptoms in line with the diagnostic criteria outlined in the Diagnostic and Statistical Manual of Mental Disorders (DSM-5)^79^. Participants are asked to rate how often they have experienced each symptom during the past two weeks on a 4-point Likert scale ranging from 0 (“not at all”) to 3 (“nearly every day”). The total score ranges from 0 to 27, with higher scores indicating greater depressive symptom severity. Although the PHQ-9 has been translated into over 20 languages^80^, and validated in numerous populations^77,81^, including cross-national contexts^82^, no formal validation study of the Hungarian version has been published to date. In the current sample, the PHQ-9 demonstrated good internal consistency (Cronbach’s α = 0.87; McDonald’s ω = 0.87).

#### Anxiety symptoms

We applied the *Generalized Anxiety Disorder Scale–7 (GAD-7)*^83^, a 7-item self-report questionnaire^78^ to assess the frequency of anxiety-related symptoms over the previous two weeks. Each item is rated on a 4-point Likert scale ranging from 0 (“not at all”) to 3 (“nearly every day”), with intermediate anchors: 1 (“several days”) and 2 (“more than half the days”). The total score ranges from 0 to 21, with higher scores indicating greater severity of anxiety symptoms. A cut-off score of 10 or above is commonly used as an indicator for clinically relevant anxiety that may warrant further evaluation for generalized anxiety disorder^58^. The GAD-7 has demonstrated strong psychometric properties (Cronbach’s α = 0.91; McDonald’s ω = 0.91), as shown in the current sample, and although it is widely used in both clinical and research settings^82,90^ a validation study in the Hungarian population has not yet been published.

In a structural model, these measures (GAD-7, PHQ-9, and DT) were used as observed indicators of a latent general psychological distress factor, capturing their shared variance rather than their individual measurement structures.

#### Emotion regulation strategies

Emotion regulation strategies were assessed using the Hungarian version of the abbreviated *Cognitive Emotion Regulation Questionnaire* (CERQ-18)^85^, validated by Miklósi et al.^86^(see also Kökönyei et al.^87^ The scale consists of 18 items, with two items per subscale, measuring nine distinct cognitive emotion regulation strategies. Five subscales represent putatively adaptive strategies (positive reappraisal, planning, acceptance, positive refocusing, putting into perspective), while four reflect putatively maladaptive strategies (self-blame, other-blame, rumination, and catastrophizing). Participants rated how frequently they typically use each strategy when experiencing negative or stressful events on a 5-point Likert scale ranging from 1 (“almost never”) to 5 (“almost always”). In the present study, two theoretically defined separate strategy types for “adaptive” (“more adaptive”) and “less adaptive” or “maladaptive” emotion regulation^14^ were used in the analyses. These second-order composite scores were calculated by averaging the corresponding first-order subscale scores. The CERQ-18 demonstrated acceptable internal consistency in the current sample, with Cronbach’s alpha coefficients of 0.77 for the adaptive subscales and 0.83 for the maladaptive subscales.

#### Automatic negative thoughts

We utilized the *Automatic Thoughts Questionnaire – Short Form (ATQ-8)*^88^, an 8-item self-report measure to assess the frequency of spontaneous negative thoughts experienced over the past two weeks (e.g., “I’m no good.”, “I feel so helpless.”, “My future is black.”). Each item is rated on a 5-point Likert scale ranging from 1 (“not at all”) to 5 (“all the time”), yielding a total score between 8 and 40, with higher scores indicating greater frequency of negative automatic thoughts. Although the ATQ-8 is not intended for diagnostic purposes, it provides a brief and psychometrically sound measure of maladaptive cognitive patterns. To reduce the impact of variability in response range and to facilitate statistical comparisons, mean item scores are often used instead of total scores^91^. For the present study, the ATQ-8 was adapted into Hungarian following established cross-cultural validation guidelines^92^. This process involved independent forward translation by two bilingual experts, followed by synthesis into a single preliminary version, then a backward translation, which was reviewed by our research team to assess conceptual and linguistic equivalence with the original English version. To ensure clarity and cultural appropriateness, a series of cognitive debriefing interviews were conducted with a small, convenient sample of respondents. Feedback was incorporated to refine item phrasing and ensure comprehensibility. Preliminary psychometric analyses in the current sample supported the internal consistency (Cronbach’s alpha was 0.90) and structural validity of the Hungarian adaptation. These results suggest that the Hungarian version of the ATQ-8 is a reliable and valid instrument for assessing negative automatic thoughts in this population.

#### Sociodemographic and clinical information

In addition to sociodemographic data participants were asked to provide information related to their cancer diagnosis and treatment. Specifically, the present study utilized four self-report items to obtain descriptive clinical information: (1) “Which part of your body was affected by the diagnosed cancer? (Please indicate the location of the primary tumor if known.)” (2) “What is the current stage classification of your illness?” (3) “What is the current status of your treatment?” and (4) “What treatments have you received for your current illness? Please indicate all types of treatment you have undergone.” For item (1), participants indicated the location of the primary tumor from a predefined list (e.g., breast, ovarian, cervical, uterine). For item (2), cancer stage was reported using standard categories: Stage 0, Stage I, Stage II, Stage III, Stage IV, remission, or “do not know.” For item (3), participants selected from the following options: before medical treatment, during medical treatment (inpatient, outpatient, or hospice), and after medical treatment. For item (4), participants selected from a predefined list of treatment types, including surgery, chemotherapy, radiotherapy, hormone therapy, immunotherapy, and other. These items were used to obtain descriptive information on cancer type and treatment status, which allowed for basic clinical characterization of the sample and were dummy coded for analysis as described below.

### Statistical analysis

Data were analyzed using IBM SPSS Statistics 28.0 (IBM Corp., Armonk, NY) and Mplus 8.0^53^. Prior to analysis, the dataset underwent systematic data cleaning to ensure quality. All 609 entries from an initial pool were reviewed for completeness, duplication, and response validity. To detect potential duplicate submissions, Internet Protocol (IP) addresses and response timestamps were examined. Entries originating from the same IP address and showing highly similar or identical response patterns were flagged for manual review. In confirmed cases of duplication, only the most complete response was retained.

Records with substantial missing data (i.e., fewer than 10% of items completed) or unrealistically short completion times (i.e., less than 100 s) were excluded from the final dataset. This data management strategy was informed by reports indicating that some participants, especially those completing the questionnaire in the hospital’s waiting rooms, were interrupted (e.g., when called in for medical appointments), leading to abrupt discontinuation. All data cleaning decisions were documented. The final analytical sample (*N* = 230) included only valid and independent responses that met the inclusion criteria for the present study, specifically female participants diagnosed with breast or gynecological cancer. In addition, cancer stage, treatment phase, and age were included as potential confounding variables and statistically controlled for in the analyses to account for their influence on psychological outcomes. Because cancer stage and treatment phase served only as a covariate, we used a pragmatic dichotomous coding (advanced vs. nonadvanced), which required grouping remission and ‘unknown’ responses with early-stage disease; this approach, while common, represents a methodological limitation. For analysis, variables were dummy coded as follows: treatment status was coded as “before treatment” (1 = yes, 0 = otherwise), “after treatment” (1 = yes, 0 = otherwise) while participants currently “during treatment” were coded 0 on both variables; cancer stage was coded as advanced (1 = stage III or IV) versus early or unknown (0 = stage 0, I, II, remission, or “do not know”). These variables were specified as exogenous predictors of the distress outcomes to statistically adjust for their potential influence. For clarity of presentation, these control paths are not depicted in the figures; however, full parameter estimates for all covariates are provided in Supplementary Tables S2a and S3a.

Prior to model estimation, standard SEM assumptions were evaluated. Univariate and multivariate distributional characteristics were inspected, and models were estimated using robust maximum likelihood (MLR) and maximum likelihood with bootstrap confidence intervals to account for deviations from normality. Linearity was examined through bivariate scatterplots, which showed no substantial departures from linear relations. Multicollinearity was assessed using the correlation matrix, with no coefficients approaching problematic levels (e.g., *r* ≥ .80). Multivariate outliers were evaluated using Mahalanobis distance (*p* < .001)^93,94^, and although several cases exceeded the critical threshold (eight cases exceeded the critical χ² value (29.9), with the largest Mahalanobis distance being 35.6), none reflected data entry errors; therefore, all cases were retained.

To address potential violations of multivariate normality, all models were additionally estimated using the robust maximum likelihood estimator (MLR). Indirect effects were evaluated using maximum likelihood with bias-corrected bootstrap confidence intervals based on 5,000 resamples to ensure stable estimation. Results obtained with MLR and with ML plus bootstrapping were highly consistent, yielding substantively similar standard errors and p-values. Full comparisons of the two estimation approaches are provided in Supplementary Tables S2a and S3a.

We conducted structural equation modeling (SEM) and path analysis using Mplus 8.10 to examine the associations between cognitive emotion regulation strategies, negative automatic thoughts, and general PD. We assessed PD through three indicators: depressive symptoms, anxiety, and cancer-specific distress. Two analytic approaches were employed to compare whether a multidimensional or a unified representation of distress provided a better fit to the data and more accurately captured the underlying psychological construct.

First, we conducted a path analysis based on observed variables, treating the three distress indicators as separate outcome variables. This model tested the direct and indirect effects of adaptive and maladaptive emotion regulation strategies on each distress indicator, including NATs as a mediator. This approach allows for comparability with prior studies that examined these outcomes separately. Due to the small sample size, we performed parameter estimation using maximum likelihood (ML) with bias‑corrected bootstrap confidence intervals based on 5,000 resamples to obtain confidence intervals for indirect effects. Missing data were handled using full information maximum likelihood (FIML). Since the model was fully saturated (i.e., all theoretically justified paths were specified), it had zero degrees of freedom and did not report model fit indices.

Second, we tested a latent variable model in which the three observed indicators served as manifest variables for a single latent factor of general psychological distress. This approach enabled us to capture the shared variance across depression, anxiety, and cancer-specific distress. Furthermore, the model permitted estimation of direct effects from NATs to individual indicators while controlling for the latent factor, analogous to a multiple indicators, multiple causes (MIMIC) model. We evaluated model fit using standard fit indices: the Comparative Fit Index (CFI), the Tucker–Lewis Index (TLI), the Root Mean Square Error of Approximation (RMSEA), and the Standardized Root Mean Square Residual (SRMR). The following criteria were used to interpret the model fit: CFI and TLI values above 0.95 indicate an excellent fit and values above 0.90 indicate an acceptable fit. RMSEA values below 0.05 indicate an excellent fit and values below 0.10 indicate an acceptable fit. SRMR values below 0.08 were considered acceptable. We estimated this model using maximum likelihood (ML) with bootstrapping (5000 resamples) and FIML to handle missing data. Modification indices were examined to determine the potential benefit of adding paths from NATs to individual distress indicators beyond the latent construct. Together, these complementary approaches provided a nuanced assessment of the shared and unique variance in distress indicators. They also allowed for an evaluation of NATs as a cognitive mediator in cancer-related psychological adaptation.

Given the cross-sectional design, we additionally estimated theoretically plausible alternative models to examine the robustness of the proposed directional assumptions. Specifically, we tested reversed path models in which NATs predicted ER strategies, which in turn predicted distress outcomes (NATs → ER → GPD). Full parameter estimates for these alternative models, as well as comparisons of information criteria (AIC, BIC), are reported in Supplementary Tables S4–S5 and Supplementary Table S6.

## Supplementary Information

Below is the link to the electronic supplementary material.

Supplementary Material 1

## Data Availability

The datasets generated and analyzed in the present study can be made available by the authors upon reasonable request.
