# Supplementary material for: Negative automatic thoughts mediate the effects of emotion regulation on distress in women with breast and gynecological cancer
Source: Sci Rep. 2026 May 29;16:18868. doi: 10.1038/s41598-026-55214-4 (PMC13276021; doi:10.1038/s41598-026-55214-4)
Supplement: Supplementary file 1 — Supplementary Material 1 [file 41598_2026_55214_MOESM1_ESM.docx]

**Supplementary Material**

*Supplementary Table S1*

**Person’s correlations between cognitive emotion regulation (CERQ-18) subscales**

|  | 1. | 2. | 3. | 4. | 5. | 6. | 7. | 8. |
| --- | --- | --- | --- | --- | --- | --- | --- | --- |
| 1. Acceptance |  |  |  |  |  |  |  |  |
| 2. Self-blame | .052 |  |  |  |  |  |  |  |
| 3. Rumination | **.146*** | **.406***** |  |  |  |  |  |  |
| 4. Positive refocusing | .122 | **–.320***** | **–.414***** |  |  |  |  |  |
| 5. Planning | **.188**** | **.148*** | **.143*** | **.138*** |  |  |  |  |
| 6. Positive reappraisal | **.288***** | –.037 | **–.253***** | **.429***** | **.332***** |  |  |  |
| 7. Putting into perspective | **.187**** | –.094 | **–.232***** | .**423***** | **.220***** | .**510***** |  |  |
| 8. Catastrophizing | .116 | **.307***** | .**851***** | **–.311***** | .073 | **–.241***** | **–.269***** |  |
| 9. Other-blame | .045 | **.301***** | **.402***** | **–.171**** | **.150*** | –.076 | –.046 | **.422***** |

*Note*. CERQ-18, Cognitive Emotion Regulation Questionnaire; **p* < .05; ***p* < .01; ****p* < .001

*Supplementary Table S2a*

**Comparison of Robust Maximum Likelihood (MLR) and ML with Bias-Corrected Bootstrapping for Standardized Path Estimates (Model 1).**

|  |  |  | Robust Maximum Likelihood (MLR) | | ML with BC Bootstrapping (5,000 draws) | | |
| --- | --- | --- | --- | --- | --- | --- | --- |
| Outcome | Predictor | β | MLR SE | MLR p | Bootstrap SE | Bootstrap p | 95% Bias-Corrected CI |
| Distress |  |  |  |  |  |  |  |
|  | Negative automatic thoughts (ATQ) | 0.294 | 0.089 | **.001** | 0.092 | **.001** | **[0.118, 0.472]** |
|  | Adaptive ER | –0.061 | 0.064 | .335 | 0.066 | .351 | [–0.192, 0.064] |
|  | Maladaptive ER | 0.196 | 0.105 | .061 | 0.104 | .059 | [–0.004, 0.398] |
|  | Age | 0.008 | 0.057 | .886 | 0.058 | .889 | [–0.107, 0.118] |
|  | After treatment | –0.094 | 0.065 | .147 | 0.065 | .147 | [–0.221, 0.033] |
|  | Before treatment | 0.045 | 0.044 | .307 | 0.045 | .316 | [–0.044, 0.134] |
|  | Cancer stage (Advanced) | –0.013 | 0.062 | .828 | 0.061 | .825 | [–0.132, 0.104] |
| PHQ |  |  |  |  |  |  |  |
|  | Negative automatic thoughts (ATQ) | 0.502 | 0.078 | **<.001** | 0.079 | **<.001** | **[0.342, 0.652]** |
|  | Adaptive ER | –0.046 | 0.046 | .324 | 0.047 | .335 | [–0.132, 0.056] |
|  | Maladaptive ER | 0.276 | 0.078 | **<.001** | 0.077 | **<.001** | **[0.123, 0.428]** |
|  | Age | 0.000 | 0.044 | .994 | 0.045 | .994 | [–0.096, 0.081] |
|  | After treatment | –0.035 | 0.048 | .458 | 0.049 | .473 | [–0.130, 0.061] |
|  | Before treatment | 0.016 | 0.038 | .679 | 0.039 | .692 | [–0.060, 0.096] |
|  | Cancer stage (Advanced) | –0.056 | 0.047 | .231 | 0.048 | .239 | [–0.149, 0.037] |
| GAD |  |  |  |  |  |  |  |
|  | Negative automatic thoughts (ATQ) | 0.370 | 0.073 | **<.001** | 0.075 | **<.001** | **[0.217, 0.513]** |
|  | Adaptive ER | –0.010 | 0.049 | .831 | 0.051 | .837 | [–0.109, 0.090] |
|  | Maladaptive ER | 0.398 | 0.075 | **<.001** | 0.074 | **<.001** | **[0.251, 0.543]** |
|  | Age | 0.017 | 0.053 | .750 | 0.054 | .756 | [–0.086, 0.127] |
|  | After treatment | –0.008 | 0.048 | .869 | 0.049 | .871 | [–0.101, 0.089] |
|  | Before treatment | 0.084 | 0.046 | .071 | 0.047 | .077 | [–0.001, 0.188] |
|  | Cancer stage (Advanced) | –0.110 | 0.048 | **.022** | 0.049 | **.025** | **[–0.208, –0.012]** |
| ATQ |  |  |  |  |  |  |  |
|  | Adaptive ER | –0.105 | 0.046 | **.021** | 0.047 | **.026** | **[–0.196, –0.009]** |
|  | Maladaptive ER | 0.708 | 0.042 | **<.001** | 0.042 | **<.001** | **[0.620, 0.784]** |
|  | Age | –0.007 | 0.048 | .891 | 0.049 | .893 | [–0.098, 0.091] |
|  | After treatment | 0.056 | 0.048 | .250 | 0.049 | .254 | [–0.036, 0.152] |
|  | Before treatment | –0.016 | 0.034 | .639 | 0.035 | .653 | [–0.084, 0.056] |
|  | Cancer stage (Advanced) | 0.073 | 0.048 | .130 | 0.048 | .132 | [–0.020, 0.170] |

*Note*. β= STDYX standardized path coefficient; SE = standard error; Bias-Corrected CI= bias-corrected confidence interval based on 5,000 bootstrap draws. MLR = Robust Maximum Likelihood estimation. MLR and ML estimation produce identical point estimates because both rely on the same maximum likelihood fitting function; however, they differ in the estimation of standard errors and significance tests. MLR provides robust standard errors and scaled test statistics that adjust for non-normality, whereas ML with bootstrapping derives empirical standard errors and confidence intervals from resampling. As shown in the table, substantive conclusions remain identical across the two estimation approaches. Maladaptive and Adaptive ER refer to composite scores of the Cognitive Emotion Regulation Questionnaire (CERQ-18).

*Supplementary Table S2b*

**Mediation Results of Emotion Regulation Strategies on Distress Outcomes through Negative Automatic Thoughts (NATs): Standardized Total, Indirect, and Direct Effects**

| Effect Type | Path | β | Bootstrap SE | Bootstrap p | 95% Bias-Corrected CI |
| --- | --- | --- | --- | --- | --- |
| Maladaptive ER |  |  |  |  |  |
| Total | Maladaptive ER → Distress | **0.405** | **0.067** | **< .001** | **[0.266, 0.525]** |
| Direct | Maladaptive ER → Distress | 0.196 | 0.104 | .059 | [–0.004, 0.398] |
| Indirect | Maladaptive ER → NATs → Distress | **0.209** | **0.067** | **.002** | **[0.085, 0.340]** |
| Total | Maladaptive ER → Depressive symptoms | **0.631** | **0.046** | **< .001** | **[0.528, 0.711]** |
| Direct | Maladaptive ER → Depressive symptoms | **0.276** | **0.077** | **< .001** | **[0.123, 0.428]** |
| Indirect | Maladaptive ER → NATs → Depressive symptoms | **0.356** | **0.062** | **< .001** | **[0.241, 0.485]** |
| Total | Maladaptive ER → Anxiety symptoms | **0.660** | **0.043** | **< .001** | **[0.563, 0.733]** |
| Direct | Maladaptive ER → Anxiety symptoms | **0.398** | **0.074** | **< .001** | **[0.251, 0.543]** |
| Indirect | Maladaptive ER → NATs → Anxiety symptoms | **0.262** | **0.054** | **< .001** | **[0.155, 0.368]** |
| Adaptive ER |  |  |  |  |  |
| Total | Adaptive ER → Distress | –0.092 | 0.068 | .175 | [–0.226, 0.039] |
| Direct | Adaptive ER → Distress | –0.061 | 0.066 | .351 | [–0.192, 0.064] |
| Indirect | Adaptive ER → NATs → Distress | –0.031 | 0.017 | .067 | [–0.074, –0.006] |
| Total | Adaptive ER → Depressive symptoms | –0.099 | 0.053 | .061 | [–0.197, 0.011] |
| Direct | Adaptive ER → Depressive symptoms | –0.046 | 0.047 | .335 | [–0.132, 0.056] |
| Indirect | Adaptive ER → NATs → Depressive symptoms | **–0.053** | **0.024** | **.029** | **[–0.106, –0.010]** |
| Total | Adaptive ER → Anxiety symptoms | –0.049 | 0.054 | .357 | [–0.155, 0.055] |
| Direct | Adaptive ER → Anxiety symptoms | –0.010 | 0.051 | .837 | [–0.109, 0.090] |
| Indirect | Adaptive ER → NATs → Anxiety symptoms | **–0.039** | **0.020** | **.050** | **[–0.085, –0.006]** |

*Note***:** β represents standardized coefficients from STDYX standardization. Maladaptive and Adaptive ER refer to composite scores of the Cognitive Emotion Regulation Questionnaire (CERQ-18). NATs refers to the Automatic Thoughts Questionnaire (ATQ-8). CI = Confidence Interval based on 5000 bootstrap resamples. All paths controlled for age, cancer stage, and treatment status.

*Supplementary Table S3a*

**Comparison of Robust Maximum Likelihood (MLR) and ML with Bias-Corrected Bootstrapping for Standardized Path Estimates (Model 2 with latent distress).**

| Outcome | Predictor | β | MLR SE | MLR p | Boot SE | Boot p | 95% Bias-Corrected CI |
| --- | --- | --- | --- | --- | --- | --- | --- |
| Latent distress* |  |  |  |  |  |  |  |
|  | Negative automatic thoughts (ATQ) | **0.536** | 0.081 | **<.001** | 0.082 | **<.001** | **[0.362, 0.686]** |
|  | Adaptive emotion regulation | –0.055 | 0.056 | .329 | 0.059 | .347 | [–0.170, 0.060] |
|  | Maladaptive emotion regulation | **0.354** | 0.088 | **<.001** | 0.085 | **<.001** | **[0.187, 0.518]** |
|  | Age | 0.009 | 0.048 | .854 | 0.051 | .854 | [–0.091, 0.109] |
|  | After treatment | –0.063 | 0.053 | .239 | 0.054 | .246 | [–0.170, 0.043] |
|  | Before treatment | 0.076 | 0.038 | .045 | 0.040 | .059 | [–0.002, 0.154] |
|  | Cancer stage (Advanced) | –0.076 | 0.049 | .122 | 0.051 | .134 | [–0.176, 0.024] |
| Negative automatic thoughts (ATQ |  |  |  |  |  |  |  |
|  | Adaptive emotion regulation | **–0.105** | 0.046 | **.021** | 0.047 | **.026** | **[–0.196, –0.009]** |
|  | Maladaptive emotion regulation | **0.708** | 0.042 | **<.001** | 0.042 | **<.001** | **[0.620, 0.784]** |
|  | Age | –0.007 | 0.048 | .891 | 0.049 | .893 | [–0.103, 0.089] |
|  | After treatment | 0.056 | 0.048 | .250 | 0.049 | .254 | [–0.038, 0.152] |
|  | Before treatment | –0.016 | 0.034 | .639 | 0.035 | .663 | [–0.085, 0.053] |
|  | Cancer stage (Advanced) | 0.073 | 0.048 | .130 | 0.048 | .132 | [–0.021, 0.169] |
| PHQ | Negative automatic thoughts (ATQ) | **0.230** | 0.109 | **.034** | 0.110 | **.037** | **[0.026, 0.456]** |
| Latent distress* | Negative automatic thoughts (ATQ) | –0.039 | 0.109 | .717 | 0.113 | 0.726 | [–0.276, 0.156] |

*Note*. * Distress was specified as a latent variable indicated by cancer-specific distress, GAD, and PHQ. β= STDYX standardized path coefficient; SE = standard error; Bias-Corrected CI= bias-corrected confidence interval based on 5,000 bootstrap draws. MLR = Robust Maximum Likelihood estimation. MLR and ML estimation produce identical point estimates because both rely on the same maximum likelihood fitting function; however, they differ in the estimation of standard errors and significance tests. MLR provides robust standard errors and scaled test statistics that adjust for non-normality, whereas ML with bootstrapping derives empirical standard errors and confidence intervals from resampling. As shown in the table, substantive conclusions remain identical across the two estimation approaches.

*Supplementary Table S3b*

**Mediation Results of Emotion Regulation Strategies on Latent Distress through Negative Automatic Thoughts (NATs): Standardized Total, Indirect, and Direct Effects**

| Effect Type | Path | β | Bootstrap SE | Bootstrap p | 95% Bias-Corrected CI |
| --- | --- | --- | --- | --- | --- |
| Maladaptive ER |  |  |  |  |  |
| Total | Maladaptive ER → Latent Distress | **0.693** | **0.048** | **< .001** | **[0.591, 0.777]** |
| Direct | Maladaptive ER → Latent Distress | **0.404** | **0.080** | **< .001** | **[0.246, 0.563]** |
| Indirect | Maladaptive ER → NATs → Latent Distress | **0.290** | **0.061** | **< .001** | **[0.175, 0.417]** |
| Adaptive ER |  |  |  |  |  |
| Total | Adaptive ER → Latent Distress | –0.072 | 0.059 | .225 | [–0.188, 0.045] |
| Direct | Adaptive ER → Latent Distress | –0.029 | 0.055 | .599 | [–0.136, 0.083] |
| Indirect | Adaptive ER → NATs → Latent Distress | **–0.043** | **0.022** | **.047** | **[–0.091, –0.006]** |

*Note***:** β represents standardized coefficients from STDYX standardization. The Latent Distress Factor is indicated by the distress thermometer, depressive symptoms (PHQ-9), and anxiety symptoms (GAD-7). Maladaptive and Adaptive ER refer to composite scores of the Cognitive Emotion Regulation Questionnaire (CERQ-18). NATs refers to the Automatic Thoughts Questionnaire (ATQ-8). CI = Confidence Interval based on 5000 bootstrap resamples. All paths controlled for age, cancer stage, and treatment status.

*Supplementary Table S4a*

**Alternative model with ML with Bias-Corrected Bootstrapping for Standardized Path Estimates (Alternative Model 1)**

| Outcome | Predictor | β | Bootstrap SE | Bootstrap p | 95% Bias-Corrected CI |
| --- | --- | --- | --- | --- | --- |
| Cancer-specific distress (DT) |  |  |  |  |  |
|  | Negative automatic thoughts (ATQ) | **0.331** | **0.091** | **<.001** | **[0.143, 0.503]** |
|  | Adaptive emotion regulation | –0.054 | 0.065 | .405 | [–0.183, 0.071] |
|  | Maladaptive emotion regulation | 0.154 | 0.101 | .125 | [–0.039, 0.354] |
|  | Age | 0.011 | 0.058 | .847 | [–0.102, 0.125] |
|  | After treatment | –0.115 | 0.066 | .081 | [–0.243, 0.013] |
|  | Before treatment | 0.052 | 0.046 | .261 | [–0.035, 0.144] |
|  | Cancer stage (Advanced) | –0.026 | 0.064 | .688 | [–0.151, 0.101] |
| Depressive symptoms (PHQ-9) |  |  |  |  |  |
|  | Negative automatic thoughts (ATQ) | **0.502** | **0.079** | **<.001** | **[0.337, 0.648]** |
|  | Adaptive emotion regulation | –0.041 | 0.049 | .402 | [–0.131, 0.062] |
|  | Maladaptive emotion regulation | **0.266** | **0.079** | **.001** | **[0.114, 0.421]** |
|  | Age | 0.013 | 0.046 | .775 | [–0.081, 0.100] |
|  | After treatment | –0.068 | 0.051 | .182 | [–0.170, 0.029] |
|  | Before treatment | 0.024 | 0.040 | .540 | [–0.050, 0.108] |
|  | Cancer stage (Advanced) | –0.060 | 0.050 | .231 | [–0.154, 0.041] |
| Anxiety symptoms (GAD-7) |  |  |  |  |  |
|  | Negative automatic thoughts (ATQ) | **0.388** | **0.076** | **<.001** | **[0.231, 0.529]** |
|  | Adaptive emotion regulation | –0.008 | 0.050 | .879 | [–0.103, 0.092] |
|  | Maladaptive emotion regulation | **0.376** | **0.074** | **<.001** | **[0.237, 0.530]** |
|  | Age | 0.022 | 0.055 | .693 | [–0.086, 0.127] |
|  | After treatment | –0.032 | 0.050 | .523 | [–0.129, 0.068] |
|  | Before treatment | 0.089 | 0.049 | .069 | [0.001, 0.192] |
|  | Cancer stage (Advanced) | **–0.131** | **0.051** | **.010** | **[–0.232, –0.032]** |
| Adaptive emotion regulation |  |  |  |  |  |
|  | Negative automatic thoughts (ATQ) | **–0.219** | **0.064** | **.001** | **[–0.345, –0.093]** |
|  | Age | –0.105 | 0.061 | .085 | [–0.224, 0.018] |
|  | After treatment | 0.027 | 0.069 | .700 | [–0.111, 0.158] |
|  | Before treatment | –0.008 | 0.070 | .911 | [–0.148, 0.102] |
|  | Cancer stage (Advanced) | –0.035 | 0.072 | .627 | [–0.181, 0.100] |
| Maladaptive emotion regulation |  |  |  |  |  |
|  | Negative automatic thoughts (ATQ) | **0.696** | **0.041** | **<.001** | **[0.609, 0.768]** |
|  | Age | –0.030 | 0.047 | .523 | [–0.119, 0.064] |
|  | After treatment | –0.038 | 0.052 | .465 | [–0.142, 0.047] |
|  | Before treatment | 0.091 | 0.060 | .127 | [–0.020, 0.211] |
|  | Cancer stage (Advanced) | –0.021 | 0.053 | .696 | [–0.121, 0.070] |

*Note*. *N* = 229. β = STDYX standardized path coefficient; SE = standard error; Bias-Corrected CI = bias-corrected confidence interval based on 5,000 bootstrap draws. Missing data were handled using Full Information Maximum Likelihood (FIML). All outcomes were modeled as observed variables. Control variables (age, treatment status, and cancer stage) were included as exogenous predictors.

*Supplementary Table S4b*

**Mediation Effects of Negative Automatic Thoughts on Distress Outcomes via Emotion Regulation Strategies**

| Effect Type | Path | β | Bootstrap SE | Bootstrap p | 95% Bias-Corrected CI |
| --- | --- | --- | --- | --- | --- |
| Outcome: PHQ–9 (Depressive Symptoms) | | | | | |
| Total Effect | ATQ → PHQ−9 | 0.696 | 0.041 | < 0.001 | [0.604, 0.767] |
| Total Indirect Effect | ATQ → ER→PHQ−9 | 0.194 | 0.058 | 0.001 | [0.085, 0.312] |
| Specific Indirect 1 | ATQ → Maladaptive ER → PHQ−9 | 0.185 | 0.057 | 0.001 | [0.079, 0.304] |
| Specific Indirect 2 | ATQ → Adaptive ER → PHQ−9 | 0.009 | 0.012 | 0.447 | [–0.012, 0.035] |
| Direct Effect | ATQ → PHQ−9 | 0.502 | 0.079 | < 0.001 | [0.337, 0.648] |
| Outcome: GAD–7 (Anxiety Symptoms) | | | | | |
| Total Effect | ATQ → GAD−7 | 0.651 | 0.041 | < 0.001 | [0.565, 0.725] |
| Total Indirect Effect | ATQ → ER→GAD−7 | 0.263 | 0.059 | < 0.001 | [0.159, 0.392] |
| Specific Indirect 1 | ATQ → Maladaptive ER → GAD−7 | 0.262 | 0.057 | < 0.001 | [0.161, 0.390] |
| Specific Indirect 2 | ATQ → Adaptive ER → GAD−7 | 0.002 | 0.011 | 0.885 | [–0.020, 0.026] |
| Direct Effect | ATQ → GAD−7 | 0.388 | 0.076 | < 0.001 | [0.231, 0.529] |
| Outcome: DT (Distress Thermometer) | | | | | |
| Total Effect | ATQ → DT | 0.450 | 0.054 | < 0.001 | [0.342, 0.551] |
| Total Indirect Effect | ATQ → ER → DT | 0.119 | 0.072 | 0.100 | [–0.017, 0.267] |
| Specific Indirect 1 | ATQ → Maladaptive ER → DT | 0.107 | 0.072 | 0.135 | [–0.028, 0.254] |
| Specific Indirect 2 | ATQ → Adaptive ER → DT | 0.012 | 0.015 | 0.436 | [–0.014, 0.047] |
| Direct Effect | ATQ → DT | 0.331 | 0.091 | < 0.001 | [0.143, 0.503] |

*Note*. *N* = 229. β = standardized path coefficients (STDYX standardization). ATQ: Automatic Thoughts Questionnaire (Negative automatic thoughts); Maladaptive ER: Maladaptive Emotion Regulation strategies (CERQ-18); Adaptive ER: Adaptive Emotion Regulation strategies (CERQ-18); PHQ-9: Depressive symptoms; GAD-7: Anxiety symptoms; DT: Distress Thermometer.95% BC CI: Bias-corrected confidence intervals based on 5,000 bootstrap resamples. The model statistically controlled for age, treatment status (before and after treatment), and cancer stage (advanced vs. early/unknown).

*Supplementary Table S5a*

**Alternative model with ML with Bias-Corrected Bootstrapping for Standardized Path Estimates (Alternative Model 2 – latent distress model)**

| Outcome | Predictor | β | Bootstrap SE | Bootstrap p | 95% Bias-Corrected CI |
| --- | --- | --- | --- | --- | --- |
| General psychological distress (Latent Factor) |  |  |  |  |  |
|  | Negative automatic thoughts (ATQ) | **0.423** | **0.082** | **<.001** | **[0.257, 0.577]** |
|  | Adaptive emotion regulation | –0.008 | 0.054 | .878 | [–0.113, 0.100] |
|  | Maladaptive emotion regulation | **0.410** | **0.082** | **<.001** | **[0.258, 0.581]** |
|  | Age | 0.024 | 0.060 | .692 | [–0.094, 0.138] |
|  | After treatment | –0.035 | 0.054 | .522 | [–0.140, 0.074] |
|  | Before treatment | 0.097 | 0.053 | .069 | [0.002, 0.211] |
|  | Cancer stage (Advanced) | **–0.143** | **0.055** | **.009** | **[–0.252, –0.035]** |
| Adaptive emotion regulation |  |  |  |  |  |
|  | Negative automatic thoughts (ATQ) | **–0.219** | **0.064** | **.001** | **[–0.345, –0.093]** |
|  | Age | –0.105 | 0.061 | .085 | [–0.224, 0.018] |
|  | After treatment | 0.027 | 0.069 | .700 | [–0.111, 0.158] |
|  | Before treatment | –0.008 | 0.070 | .911 | [–0.148, 0.123] |
|  | Cancer stage (Advanced) | –0.035 | 0.072 | .627 | [–0.181, 0.100] |
| Maladaptive emotion regulation |  |  |  |  |  |
|  | Negative automatic thoughts (ATQ) | **0.696** | **0.041** | **<.001** | **[0.609, 0.768]** |
|  | Age | –0.030 | 0.047 | .523 | [–0.119, 0.064] |
|  | After treatment | –0.038 | 0.052 | .465 | [–0.142, 0.063] |
|  | Before treatment | 0.091 | 0.060 | .127 | [–0.020, 0.211] |
|  | Cancer stage (Advanced) | –0.021 | 0.053 | .696 | [–0.121, 0.087] |
| Depressive symptoms (PHQ-9) direct paths |  |  |  |  |  |
|  | Negative automatic thoughts (ATQ) | 0.179 | 0.101 | .076 | [–0.036, 0.357] |
|  | Adaptive emotion regulation | –0.034 | 0.043 | .423 | [–0.118, 0.052] |
|  | Maladaptive emotion regulation | –0.048 | 0.087 | .580 | [–0.238, 0.107] |
| Cancer-specific distress (DT) direct paths |  |  |  |  |  |
|  | Negative automatic thoughts (ATQ) | –0.025 | 0.105 | .808 | [–0.231, 0.180] |
|  | Adaptive emotion regulation | –0.047 | 0.060 | .433 | [–0.164, 0.072] |
|  | Maladaptive emotion regulation | –0.191 | 0.099 | .054 | [–0.398, –0.006] |
| Factor Loadings (Latent Factor F) |  |  |  |  |  |
|  | Distress Thermometer (DT) | **0.843** | **0.113** | **<.001** | **[0.614, 1.064]** |
|  | Depressive symptoms (PHQ-9) | **0.766** | **0.114** | **<.001** | **[0.565, 1.017]** |
|  | Anxiety symptoms (GAD-7) | **0.917** | **0.026** | **<.001** | **[0.863, 0.965]** |

*Note*. *N* = 229; β = STDYX standardized path coefficient; SE = standard error; Bias-Corrected CI = bias-corrected confidence interval based on 5,000 bootstrap draws. Missing data were handled using Full Information Maximum Likelihood (FIML). General psychological distress was modeled as a latent factor with DT, PHQ-9, and GAD-7 as indicators. Direct paths were estimated for DT and PHQ-9 to account for residual variance not captured by the latent factor. Control variables (age, treatment status, and cancer stage) were included as exogenous predictors.

*Supplementary Table S5b*

**Standardized Total, Indirect, and Direct Effects of Negative Automatic Thoughts (ATQ-8) on Latent General Psychological Distress through Cognitive Emotion Regulation Strategies (Alternative Model**)

| Effect Type | Path | β | Bootstrap SE | Bootstrap p | 95% Bias-Corrected CI |
| --- | --- | --- | --- | --- | --- |
| Total Effect | ATQ → Latent Distress | 0.710 | 0.046 | < 0.001 | [0.618, 0.794] |
| Total Indirect Effect | ATQ → ER Strategies → Latent Distress | 0.287 | 0.065 | < 0.001 | [0.174, 0.434] |
| Specific Indirect 1 | ATQ → Maladaptive ER → Latent Distress | 0.285 | 0.063 | < 0.001 | [0.179, 0.431] |
| Specific Indirect 2 | ATQ → Adaptive ER → Latent Distress | 0.002 | 0.012 | 0.884 | [-0.021, 0.029] |
| Direct Effect | ATQ → Latent Distress | 0.423 | 0.082 | < 0.001 | [0.257, 0.577] |

*Note*. *N* = 229. Values presented are standardized STDYX coefficients. The model was estimated using Maximum Likelihood (ML) with 5,000 bootstrap resamples to derive p-values and confidence intervals for indirect effects. Latent General Psychological Distress is a latent factor indicated by the Distress Thermometer (DT), PHQ-9, and GAD-7. Clinical control variables (age, cancer stage, and treatment status) were included in the model but are omitted from the table for clarity. ATQ: ER= emotion regulation. Negative automatic thoughts.

*Supplementary Table S6*

**Model fit indices of the models**

| Modell | Estimation | χ2 | df | p | CFI | TLI | RMSEA | SRMR | AIC | BIC |
| --- | --- | --- | --- | --- | --- | --- | --- | --- | --- | --- |
| Path analysis model | MLR | 0.000 | 0 | - | 1.000 | 1.000 | 0.000 | 0.000 | 4850.58 | 4981.23 |
| Path analysis model | ML/Boot | 0.000 | 0 | - | 1.000 | 1.000 | 0.000 | 0.000 | 4850.58 | 4981.23 |
| Alternative path analysis | ML/Boot | 0.000 | 0 | - | 1.000 | 1.000 | 0.000 | 0.000 | 6256.00 | 6410.51 |
| Latent distress SEM model | MLR | 14.782 | 12 | 0.254 | 0.996 | 0.989 | 0.032 | 0.026 | 4840.66 | 4930.05 |
| Latent distress SEM model | ML/Boot | 14.080 | 12 | 0.296 | 0.997 | 0.993 | 0.027 | 0.026 | 4840.66 | 4930.05 |
| Alternative latent distress SEM model | ML/Boot | 0.000 | 0 | - | 1.000 | 1.000 | 0.000 | 0.000 | 6256.00 | 6410.51 |

*Note.* The identical AIC and BIC values in Rows 3 and 6 reflect the fact that both models were saturated, had zero degrees of freedom, and estimated the same number of free parameters. As a result, they yielded the same loglikelihood and reproduced the observed covariance matrix perfectly. Therefore, these fit indices are not informative for distinguishing between the two models.
